# Intracellular antibody immunity and its applications

**DOI:** 10.1371/journal.ppat.1008657

**Published:** 2020-08-06

**Authors:** Jingwei Zeng, Leo C. James

**Affiliations:** 1 University of Cambridge School of Clinical Medicine, Cambridge, United Kingdom; 2 The Medical Research Council Laboratory of Molecular Biology, Cambridge, United Kingdom; University of Michigan Medical School, UNITED STATES

## What is intracellular antibody immunity?

Antibodies or immunoglobulins (Ig) are proteins secreted into the extracellular space by B cells to bind to pathogens and antigens. In doing so, they can prevent infection, neutralize toxins, and stimulate the immune response. Antibodies achieve these effector functions by activating other serum proteins, such as complement, or by engaging antibody receptors expressed on the surface of professional immune cells. Importantly, these processes all take place outside cells. Antibodies are also internalized by cells and engage with receptors expressed in endosomal compartments, such as the neonatal fragment crystallizable region (Fc) receptor and the polymeric Ig receptor. These receptors have roles in recycling and transcytosis—the redistribution of antibodies back into serum or transport through the cell to specific tissue compartments such as the gut epithelium or from mother to fetus. In the last decade, however, it has been discovered that antibodies have a second life inside the cytosol, where they engage with a specialized antibody receptor called tripartite motif-containing protein (TRIM) 21 and activate a second line of immune defense [1].

## What is TRIM21?

TRIM21 is a ubiquitously expressed, type I interferon–inducible cytosolic protein that binds to antibodies with high affinity [2,3]; indeed, TRIM21 is the highest affinity IgG receptor in humans [1]. Like other members of the TRIM family, TRIM21 contains a RING-type E3 ubiquitin ligase domain followed by a B-box domain and a coiled-coil domain that is thought to form an antiparallel homodimer [4]. TRIM21 also contains a C-terminal PRYSPRY domain, the 2 copies of which allow simultaneous binding of the 2 heavy-chains found in an antibody [3]. TRIM21 binds to all 4 subclasses of IgG (IgG1, IgG2, IgG3, and IgG4) with comparable affinities, and this binding is remarkably highly conserved, meaning that human and mouse TRIM21 will bind to antibodies from other mammals [2]. In addition, TRIM21 has also been shown to bind to the heavy-chains of IgA and IgM, albeit weaker than IgG [5]. This is in contrast to classical cell surface antibody receptors, which are completely unrelated to TRIM21 and display strong selectivity for specific antibody isotype and subclass.

## What does TRIM21 do?

**Fig 1:**
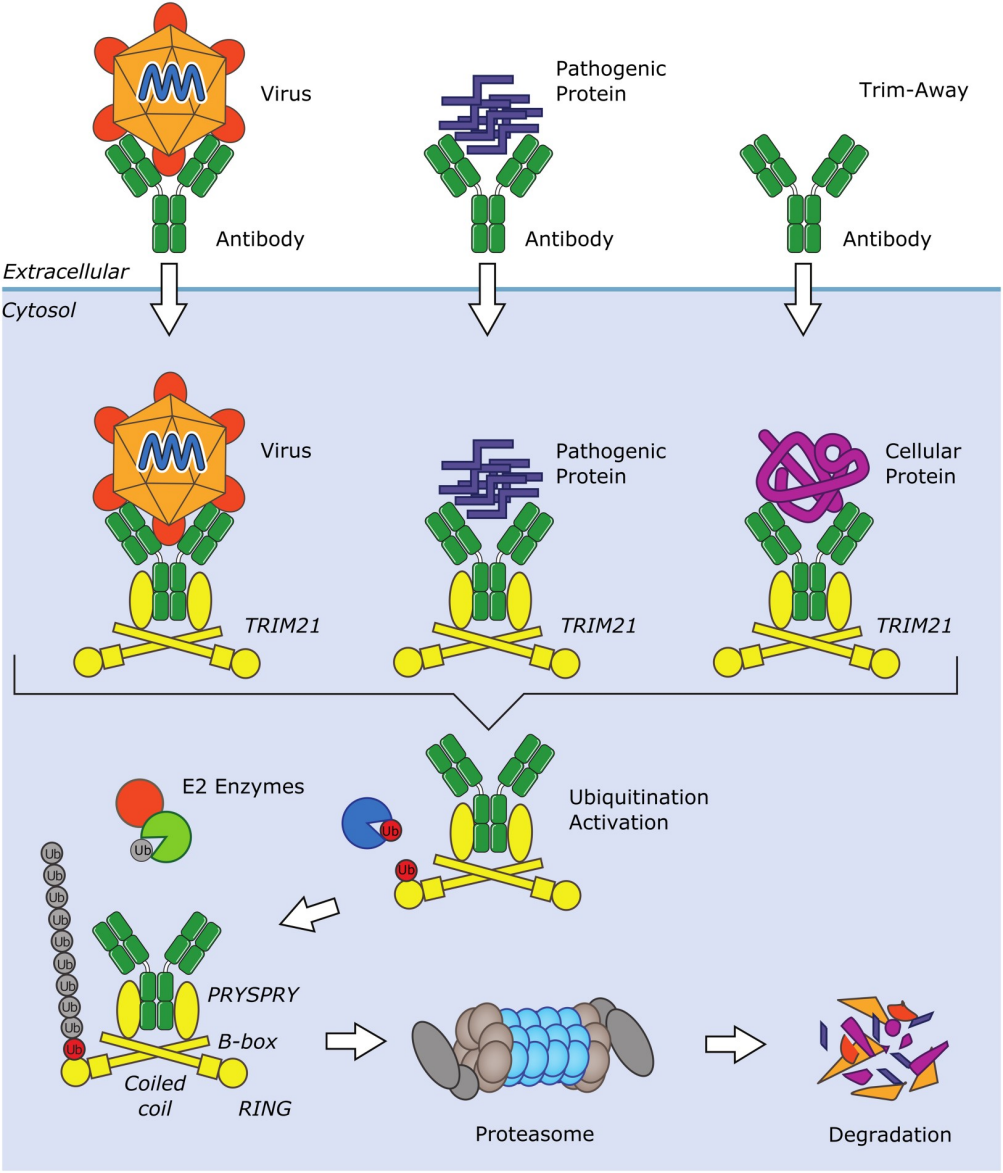
Schematic overview of TRIM21-mediated degradation of pathogens and proteins. *Image credit*: *Visual Aids Department*, *MRC Laboratory of Molecular Biology*. TRIM21, tripartite motif-containing protein 21.

Antibodies don’t normally access the cytosol because they can’t pass through plasma or endosomal membranes. However, they are good at opsonizing (binding to) viruses in the extracellular space. Viruses are obligate intracellular pathogens that have evolved specific mechanisms to trigger endocytosis and disrupt endosomal membranes in order to gain access to cellular machinery. An antibody-bound virus that escapes the endosomal compartment and enters the cytosol during infection will be met by TRIM21, which detects the virus by binding to the antibody Fc region. Importantly, as well as being an antibody receptor, TRIM21 is capable of catalyzing ubiquitination using its RING domain [1,6]. Once TRIM21 detects an antibody-bound virus, it becomes activated and begins synthesizing ubiquitin chains. These chains have 2 functions: They cause proteasomal degradation of the virus, and they stimulate immune signaling (Fig 1). This combination of sensor and effector responses provides both an immediate countermeasure against the virus and activates an ongoing antiviral state throughout the host. Therefore, TRIM21 provides a crucial mechanism by which non–entry blocking antibodies deposited on the surface of viral particles can mediate a post-entry inhibition to viral replication. For instance, the humoral response to human adenovirus 5 (AdV5) predominantly generates non–entry blocking antibodies directed against the viral hexon protein [7], meaning that AdV5 bound by this antibody can still engage cellular receptors and enter cells by endocytosis [8]. Nevertheless, this non–entry blocking anti-hexon antibody has been shown to mediate TRIM21-dependent post-entry neutralization of AdV5 [8].

## How does TRIM21 work?

When the RING domain of TRIM21 is activated, it begins catalyzing the assembly of self-anchored, K63-linked ubiquitin chains in conjunction with the E2 enzymes Ube2W and Ube2N/2V2 [6]. These self-anchored K63-linked ubiquitin chains are subsequently modified by additional ubiquitin chains with a K48-linkage [6]. TRIM21-mediated ubiquitination results in the recruitment of proteasomes and degradation of the antibody-bound viral particles [1]. This proteasome-mediated degradation of virus-antibody complexes is a remarkably fast process, and in the case of AdV5, it is facilitated by the cofactor AAA-ATPase valosin-containing protein (VCP)/p97 and allows TRIM21 to destroy an incoming virus before it begins replicating [1]. However, VCP is not always required for TRIM21-mediated degradation of simpler substrates, such as IgG Fc protein expressed inside cells [9].

TRIM21-mediated antibody-dependent intracellular neutralization (ADIN) is an incredibly efficient process; just 2 antibody molecules per adenovirus particle can be sufficient for post-entry neutralization of adenovirus in cultured cells [10]. ADIN of nonenveloped viruses has been demonstrated in a diverse range of cell lines from a variety of mammalian species as well as in in vivo mouse models [8,11]. Most recently, swine TRIM21 has been shown to mediate antiserum-dependent neutralization of the foot-and-mouth disease virus [12]. In addition to virus neutralization, TRIM21 mediates antibody-dependent inhibition of intercellular seeding of tau aggregates [13]. Similar to virus neutralization, the inhibition of tau seeding is both VCP and proteasome dependent [13] (Fig 1).

When TRIM21 detects antibody-coated pathogens inside the cell, it also triggers innate immune signaling pathways, including NF-κB, AP-1, IRF3, IRF5, and IRF7 [14]. TRIM21 can do this because the K63-linked ubiquitin chains it synthesizes are potent immune activators. Nuclear factor-κB (NF-κB) is a master regulator of innate immune signaling, and K63-linked ubiquitin chains are sufficient for NF-κB pathway activation [15]. TRIM21 has been shown to activate NF-κB upon infection with antibody-bound human adenovirus and rhinovirus, feline calicivirus, and *Salmonella enterica* [14,16]. Importantly, TRIM21 synergizes with other pattern-recognition receptors to potentiate immune sensing. When TRIM21 causes the proteasomal degradation of an incoming virus, it exposes the viral genome to cytosolic nucleic acid sensors. TRIM21 has been shown to reveal the genome of adenovirus to cGAS/STING and the genome of rhinovirus to RIG-I/MAVS [16]. In primary human macrophages, TRIM21-mediated viral genome exposure stimulates a cascade of sensors ultimately leading to activation of the inflammasome, pyroptosis, and the release of IL-1β [17]. Unlike nonimmune cells, macrophages express a variety of Fcγ receptors in addition to TRIM21, and in these cells, the Fcγ receptors were shown to contribute to viral neutralization by targeting antibody-virus complexes for destruction in the phagolysosome compartment [18]. However, even in these Fcγ-expressing professional immune cells, TRIM21 acts as an important safety mechanism to destroy any antibody-coated viruses that escape into the cytosol, and virus neutralization is only impaired when both of these pathways are suppressed [17].

By targeting antibody-coated virus particles for proteasomal degradation, TRIM21-mediated ADIN can, in theory, generate peptide antigens for presentation on major histocompatibility complex (MHC) class I molecules via the classical antigen presentation pathway. In professional antigen-presenting cells, the viral antigens can also be presented on MHC class II molecules through the cross-presentation pathway. Recently, a mutated anti-adenovirus IgG with increased affinity for TRIM21 was shown to enhance dendritic cell (DC) activation and cytokine secretion upon infection of DCs with mutant IgG complexed adenovirus [19]. In addition, DCs primed with mutant IgG-adenovirus complex were shown to stimulate CD8^+^ T-cell proliferation and cytokine release in a co-culture system [19].

One major limitation of this TRIM21-mediated ADIN mechanism is the requirement for the virus to carry antibodies with it into the cytosol. Although this is frequently the case for nonenveloped viruses that enter the cell by triggering receptor-mediated endocytosis, it can be circumvented by enveloped viruses that shed their outer lipid envelope during host cell entry.

## How can we exploit TRIM21?

The key feature of TRIM21-mediated intracellular antibody immunity is that TRIM21 does not directly engage with its target but with target-bound antibodies. This explains why it works against such a diverse range of targets including viruses, bacteria, and proteopathic agents. Moreover, it means that TRIM21 will target for degradation any antibody-bound protein in the cytosol. This property can be exploited to carry out protein depletion in living cells (Fig 1). In the technology Trim-Away, antibodies against endogenous cellular proteins are delivered into cells by methods such as electroporation or microinjection [9]. The resulting antibody-bound target is recognized by TRIM21, and the entire protein complex is degraded by the ubiquitin-proteasome system [9]. The cross-species activity of TRIM21 is particularly helpful here as it enables off-the-shelf antibodies raised in different mammals to be used in degradation experiments without the need for modification [9]. Trim-Away provides an alternative to small interfering RNA (siRNA) and can be used in the same kind of experiments to study protein function. However, because Trim-Away works at the protein level, it is much faster and will remove a target protein within hours [9], rather than days as with siRNA. Trim-Away also has 3 further benefits. First, it enables the depletion of proteins in cells that are not amenable to standard genetic-based techniques, such as in primary cells in which active nucleotide-sensing pathways generate unwanted inflammatory responses to DNA or RNA transfection [20]. Indeed, Trim-Away has been used to demonstrate that NLRP3 is vital for inflammasome formation and interleukin-1β secretion by ex vivo human monocyte–derived macrophages [9]. Second, Trim-Away can be used in cells that are transcriptionally quiescent, such as oocytes, in which the target protein has a long cytosolic half-life, or to acutely remove proteins that are essential for long-term cell survival. For example, Trim-Away was used to rapidly degrade target proteins at specific stages of cell division during oocyte meiosis [21]. Third, Trim-Away allows transcriptional pathways to be rapidly activated. For instance, Trim-Away has been used to degrade IκB and induce NF-κB activation [9]. Although transient selective protein degradation is a distinguishing feature of Trim-Away, it can also be used to achieve long-lasting persistent protein knockdown by expressing nanobody-Fc fusion proteins [9]. Expression of a nanobody-Fc fusion by mRNA transfection has been shown to allow targeting of nuclear-localized proteins such as histone H2B for degradation, whereas full-length antibodies are too large to access the nuclear compartment [9].

## What next?

Understanding intracellular antibody immunity and the role of TRIM21 has potential implications for future vaccine and gene therapy design. TRIM21 was shown to be a major contributing factor for inhibiting adenoviral gene delivery in vivo in the presence of preexisting antibody [8]. Preventing this, for instance, by using a small molecule inhibitor of TRIM21, could make repeated viral-based gene therapy treatments viable. Conversely, activating TRIM21 during vaccination could help induce a more robust protective response. This is because TRIM21-mediated proteasomal degradation of viral proteins has the potential to generate peptides for antigen presentation [19], and TRIM21 activation of innate signaling could provide costimulatory signals for professional immune cells. Additionally, there may be ways to expand Trim-Away from a laboratory tool to a therapeutic modality. Trim-Away has been shown to function in living organisms and has been used to study embryogenesis in zebra fish [22]. This was possible despite the fact that TRIM21 is naturally only found in mammals because the ubiquitin-proteasome system that Trim-Away relies on is highly conserved across eukaryotes. In this example, both the TRIM21 and antibody proteins were introduced by microinjection. Therapeutic delivery of antibodies into the cells of complex living organisms is still far off, as the same barriers that ordinarily keep antibodies outside the cell also prevent easy delivery of protein-based drugs into the cytosol. One possible solution may be to replace antibodies with a bispecific small molecule, such as a bispecific proteolysis-targeting chimera (PROTAC). PROTACs recruit E3 ubiquitin ligases to degrade intracellular proteins [23], and TRIM21 may be ideally suited for such an approach, given it already uses an intermediary molecule for target recruitment. However, such technologies require comprehensive understanding of TRIM21 structure, mechanism of regulation, activation and enzymatic catalysis, which are the subject of current and future studies.
